# Supplementary material for: Modeling glioblastoma heterogeneity as a dynamic network of cell states
Source: Mol Syst Biol. 2021 Sep 16;17(9):e10105. doi: 10.15252/msb.202010105 (PMC8444284; doi:10.15252/msb.202010105)
Supplement: Supplementary file 6 — Source Data for Figure 5 [file MSB-17-e10105-s004.zip › Figure5A_sourcedata/GSEA_3071/hallmarks_stateII.GseaPreranked.1623742536692/HALLMARK_ADIPOGENESIS.html]

Details for gene set HALLMARK\_ADIPOGENESIS[GSEA]

|  || Dataset | state23071 |
| Phenotype | NoPhenotypeAvailable |
| Upregulated in class | na\_neg |
| GeneSet | HALLMARK\_ADIPOGENESIS |
| Enrichment Score (ES) | -0.15990585 |
| Normalized Enrichment Score (NES) | -0.77167225 |
| Nominal p-value | 0.78900707 |
| FDR q-value | 0.92178804 |
| FWER p-Value | 1.0 |
Table: GSEA Results Summary

  

Fig 1: Enrichment plot: HALLMARK\_ADIPOGENESIS      
 Profile of the Running ES Score & Positions of GeneSet Members on the Rank Ordered List

  

| PROBE | GENE SYMBOL | GENE\_TITLE | RANK IN GENE LIST | RANK METRIC SCORE | RUNNING ES | CORE ENRICHMENT || 1 | COL4A1 |  |  | 168 | 0.929 | -0.0055 | No |
| 2 | MGLL |  |  | 227 | 0.848 | 0.0130 | No |
| 3 | CAVIN1 |  |  | 339 | 0.719 | 0.0134 | No |
| 4 | CAVIN2 |  |  | 454 | 0.649 | 0.0104 | No |
| 5 | HADH |  |  | 706 | 0.530 | -0.0311 | No |
| 6 | SLC1A5 |  |  | 757 | 0.514 | -0.0236 | No |
| 7 | RAB34 |  |  | 803 | 0.496 | -0.0155 | No |
| 8 | ME1 |  |  | 835 | 0.489 | -0.0042 | No |
| 9 | GADD45A |  |  | 867 | 0.480 | 0.0068 | No |
| 10 | SSPN |  |  | 1081 | 0.421 | -0.0296 | No |
| 11 | UCP2 |  |  | 1138 | 0.410 | -0.0275 | No |
| 12 | GPD2 |  |  | 1202 | 0.397 | -0.0277 | No |
| 13 | AK2 |  |  | 1378 | 0.360 | -0.0570 | No |
| 14 | IMMT |  |  | 1436 | 0.350 | -0.0576 | No |
| 15 | NABP1 |  |  | 1443 | 0.349 | -0.0455 | No |
| 16 | MRPL15 |  |  | 1477 | 0.343 | -0.0404 | No |
| 17 | SLC5A6 |  |  | 1503 | 0.340 | -0.0334 | No |
| 18 | GRPEL1 |  |  | 1531 | 0.335 | -0.0271 | No |
| 19 | STOM |  |  | 1594 | 0.325 | -0.0298 | No |
| 20 | COL15A1 |  |  | 1798 | 0.299 | -0.0684 | No |
| 21 | DNAJC15 |  |  | 1812 | 0.298 | -0.0601 | No |
| 22 | COQ3 |  |  | 1909 | 0.284 | -0.0728 | No |
| 23 | CYC1 |  |  | 1938 | 0.281 | -0.0689 | No |
| 24 | ESYT1 |  |  | 2052 | 0.267 | -0.0865 | No |
| 25 | SOWAHC |  |  | 2073 | 0.265 | -0.0812 | No |
| 26 | RMDN3 |  |  | 2120 | 0.261 | -0.0824 | No |
| 27 | ARL4A |  |  | 2167 | 0.255 | -0.0839 | No |
| 28 | SLC19A1 |  |  | 2206 | 0.252 | -0.0836 | No |
| 29 | PHYH |  |  | 2249 | -0.254 | -0.0841 | No |
| 30 | POR |  |  | 2321 | -0.261 | -0.0915 | No |
| 31 | CAT |  |  | 2344 | -0.264 | -0.0867 | No |
| 32 | AGPAT3 |  |  | 2375 | -0.267 | -0.0838 | No |
| 33 | COX7B |  |  | 2400 | -0.271 | -0.0792 | No |
| 34 | RNF11 |  |  | 2520 | -0.288 | -0.0975 | No |
| 35 | TOB1 |  |  | 2580 | -0.298 | -0.1006 | No |
| 36 | DNAJB9 |  |  | 2618 | -0.303 | -0.0979 | No |
| 37 | COX6A1 |  |  | 2629 | -0.305 | -0.0886 | No |
| 38 | SORBS1 |  |  | 2639 | -0.307 | -0.0789 | No |
| 39 | NDUFAB1 |  |  | 2716 | -0.320 | -0.0853 | No |
| 40 | NDUFB7 |  |  | 2852 | -0.345 | -0.1053 | No |
| 41 | ECH1 |  |  | 2885 | -0.353 | -0.0996 | No |
| 42 | ELOVL6 |  |  | 2958 | -0.366 | -0.1032 | No |
| 43 | ACOX1 |  |  | 3121 | -0.404 | -0.1276 | No |
| 44 | LAMA4 |  |  | 3193 | -0.426 | -0.1286 | No |
| 45 | DECR1 |  |  | 3261 | -0.444 | -0.1280 | No |
| 46 | BAZ2A |  |  | 3385 | -0.492 | -0.1394 | No |
| 47 | UQCR11 |  |  | 3469 | -0.527 | -0.1395 | Yes |
| 48 | PHLDB1 |  |  | 3480 | -0.533 | -0.1213 | Yes |
| 49 | BCL6 |  |  | 3542 | -0.566 | -0.1144 | Yes |
| 50 | CCNG2 |  |  | 3650 | -0.648 | -0.1158 | Yes |
| 51 | VEGFB |  |  | 3726 | -0.705 | -0.1070 | Yes |
| 52 | IDH1 |  |  | 3842 | -0.832 | -0.1032 | Yes |
| 53 | APOE |  |  | 3864 | -0.860 | -0.0750 | Yes |
| 54 | APLP2 |  |  | 3912 | -0.967 | -0.0491 | Yes |
| 55 | GPX4 |  |  | 3915 | -0.986 | -0.0114 | Yes |
| 56 | ITGA7 |  |  | 4030 | -1.454 | 0.0168 | Yes |
Table: GSEA details [plain text format]

  

Fig 2: HALLMARK\_ADIPOGENESIS: Random ES distribution      
 Gene set null distribution of ES for **HALLMARK\_ADIPOGENESIS**
